# Bilateral papillary oedema – case report

**Published:** 2018

**Authors:** Cristina Culea, Bogdana Tăbăcaru, Tudor Horia Stanca

**Affiliations:** * Ophthalmology Clinic, “Agrippa Ionescu” Clinical Emergency Hospital, Bucharest, Romania; ** Department of Ophthalmology, “Carol Davila” University of Medicine and Pharmacy, Bucharest, Romania

**Keywords:** high blood pressure, hypertensive neuroretinopathy, papillary oedema, pheochromocytoma, young age

## Abstract

Objective: We report one case of malignant high blood pressure with no systemic signs but with ocular complaints.

Methods: The paper presents the case of a 31-year-old male who complained of sudden loss of visual acuity in both eyes. The ophthalmological examination revealed bilateral papillary oedema. General and cardiological examinations revealed he was suffering from malignant high blood pressure.

Results: The pathogenic treatment resulted in resolution of signs and symptoms with favourable ophthalmological evolution and almost entirely functional recovery. Clinical, paraclinical and imagistic data suggested the diagnosis of pheochromocytoma.

Conclusion: This case highlighted ocular complications of high blood pressure. The paper summarizes the differential diagnosis and management of high blood pressure and reviews the most common causes of secondary hypertension in young patients.

## Introduction

High blood pressure is a major cause of worldwide mortality. According to SEPHAR study, the prevalence of primary and secondary high blood pressure in Romania is 40% [**1**]. In Romania, there are no studies regarding the prevalence of hypertensive neuroretinopathy, but, doubtless, their existence would be necessary.

There are three important factors that play a key role in hypertensive retinopathy pathogenesis [**2**]: 

- **Vasoconstriction:**

o The first response of retinal arterioles to increased blood pressure is the narrowing of the lumen (vasoconstriction), which is proportional to the severity of hypertension. Over time, elevated pressure damages vascular endothelium. Degeneration of smooth muscle fibers in the vascular wall leads to breaks and leakage of plasma into the vessel wall; plasma clotting within the vessel wall produce mural thickening and luminal narrowing, process called fibrinous necrosis [**3**]. Arteriosclerosis is irreversible and does not regress under treatment. It occurs both in young individuals and in the elderly, who suffer from pre-existing vascular involutional sclerosis.

- **Arteriosclerosis**

o It is manifested as changes in arteriolar reflex and arteriovenous narrowing due to thickening of the vessels wall and it reflects the long duration of the hypertension. In older patients, arteriosclerotic changes may preexist due to involutional sclerosis.

- **Increased vascular permeability**

o Switching from hypertensive angiopathy to retinopathy is confronted with rupture of the blood-retinal barrier. The blood-retinal barrier consists of vascular endothelial cells and pigmented epithelial cells connected by tight junctions that form a selective barrier, restricting ions transfer, substrate and water permeability, allowing a proper neuronal function. Therefore, there are two components [**4**]:

- Inner barrier (retinal vascular endothelium) – tight junctions between capillary endothelial cells

- Outer barrier (retinal pigmented epithelium) – tight junctions between pigmentary epithelial cells

o In hypertensive retinopathy, the outer barrier is spared, while the inner barrier is not. It is responsible for [**5**-**7**]:

- Retinal bleeding – due to altered vascular artery wall. They are superficial, flame shaped and located in the nerve fibre layer.

- Exudates:

• Cotton wool spots – areas of ischemia in the nerve fibre layer. Ischemia to the nerve fibres leads to decreased axoplasmic flow, nerve swelling and ultimately fluffy opacification [**3**]. They are superficial, perivascular and respect the macula.

• Hard exudates – occur as a result of lipid accumulations in haemorrhagic areas. They have a macular radial disposition around the fovea, giving the appearance of the macular star. 

- Papillary oedema – due to leakage and ischemia of arterioles supplying the optic disc that undergo necrosis. The ischemia causes swelling and blurred disc margins and the leakage causes peripapillary haemorrhage and disc oedema [**3**]. The presence of papillary oedema is manifested by the decrease of the visual acuity and requires immediate antihypertensive treatment.

Classification [**8**] of retinal manifestations in high blood pressure:

1. Hypertensive angiopathy – includes angiosclerosis with its four stages (it may pre-exist in elderly)

Retinal angiosclerosis [**6**]:

• Stage I – subtle broadening of the arteriolar light reflex, mild generalized arteriolar attenuation, particularly of small branches, and vein concealment.

• Stage II – obvious broadening of arteriolar light reflex and deflection of veins at arteriovenous crossings (Salus sign).

• Stage III – copper-wiring of arterioles, banking of veins distal to arteriovenous crossings (Bonnet sign), tapering of veins on either side of the crossings (Gunn sign) and right-angled deflection of veins.

• Stage IV – silver-wiring of arterioles and stage III changes.

2. Hypertensive retinopathy – it is characterised by:

• Arteriolar constriction

o Focal

o Generalized

o Arteriosclerosis (arteriovenous changes)

• Extravascular signs

o Flame shaped haemorrhage

o Cotton wool spots and macular star

3. Hypertensive neuroretinopathy – it is characterised by:

• Hypertensive retinopathy

• Optic disc oedema

Patients are generally asymptomatic in early stages and have bilateral ophthalmoscopic changes. If papillary and macular oedema occurs, in later stages, the patient complains of visual acuity loss [**5**]. Patient evaluation begins with visual acuity testing and examination of the anterior and posterior segment using the biomicroscope. An optical coherence tomography (OCT) is necessary in order to evaluate the severity of retinal damage. Also, a fluorescein angiography could be necessary to discriminate between ischemic macular disorders, which cannot be evaluated by OCT, and non-ischemic macular disorders. Finally, a complete anamnesis and referral to other medical specialities, like cardiology, endocrinology, internal medicine, etc. is essential.

Differential diagnosis includes diabetic retinopathy, retinal vein occlusion, anaemia, syphilis, irradiation retinopathy and papillary oedema caused by intracranial high pressure [**4**].

The treatment’s purpose is an optimum diet and weight, a normal value of the blood pressure by referral to the cardiologist and regular ophthalmoscopic examinations to follow retinal changes [**5**].

Without treatment, the general prognosis is difficult to assess, because a number of systemic complications may occur over time: stroke, acute myocardial infarction, heart failure, rhythm disorders (atrial fibrillation, ventricular extrasystoles and ventricular tachycardia), chronic kidney disease, peripheral vascular disease [**9**]. Ophthalmologic prognosis depends on the evolution of the underlying disease. High blood pressure can induce numerous retinal severe complications: retinal vein occlusion, retinal artery occlusion, retinal aneurysms, decrease of visual acuity in time due to lesions in the optic nerve and macula. Also, high blood pressure can determine: intravitreal bleeding, oculomotor paralysis by stroke, horizontal diplopia due to sixth nerve palsy caused by stretching of one or both abducens nerves over the petrous tip because of the papillary oedema or hemianopathic deficiencies by affecting intracranial optic nerves pathways [**6**].

The ophthalmological prognosis is good with the treatment of the high blood pressure, although the general prognosis remains uncertain [**5**].

## Material and methods – Case Report

A 31-year-old male presented to the ophthalmological department complaining of sudden loss of visual acuity in both eyes and metamorphopsia in the right eye for the past two weeks. Anamnesis revealed he had been suffering from high blood pressure for approximately three years and that he did not follow the given treatment at all, meaning nebivolol and a fixed combination of perindopril with indapamide. The patient also presented profuse diaphoresis and anxiety. 

At presentation, the ophthalmological examination revealed a visual acuity of 20/ 25 S.C. (ROD = +0.25 SPH) in the right eye and 20/ 20 S.C. (ROS = + 0.75 SPH ˆ -0.50 CYL ˆ 6˚) in the left eye. Intraocular pressure was 18 mmHg GAT in his right eye and 19 mmHg GAT in his left eye.

The general examination was within normal limits, except for the blood pressure, which was 180/ 100 mmHg.

**Fig. 1:**
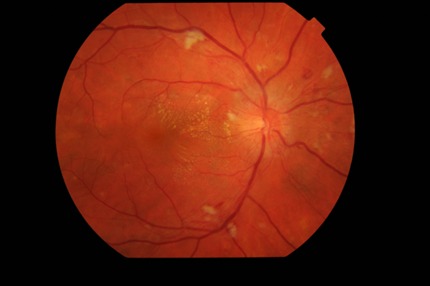
Ophthalmoscopic examination of the right eye

**Fig. 2:**
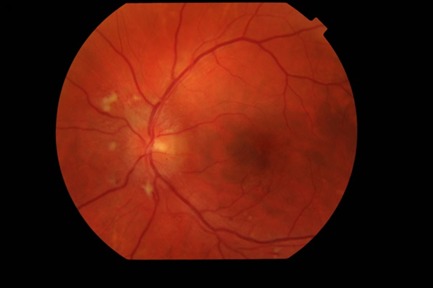
Ophthalmoscopic examination of the left eye

The biomicroscopic examination revealed a normal appearance of the anterior segment in both eyes. The fundus examination of the right eye (**Fig. 1**) revealed blurred margins, elevated and strongly hyperemic papilla, arteries with irregular caliber, turgescent veins, arteriovenous crosses with vein deflections, cotton wool spots located parallel to the temporal arches and a particular aspect called macular star, involving the presence of numerous hard exudates in the Henle fibers. The fundus examination of the left eye (**Fig. 2**) revealed similar changes of the papilla and vessels, the presence of peripapillary cotton wool spots respecting the papilla and no macular changes.

**Fig. 3:**
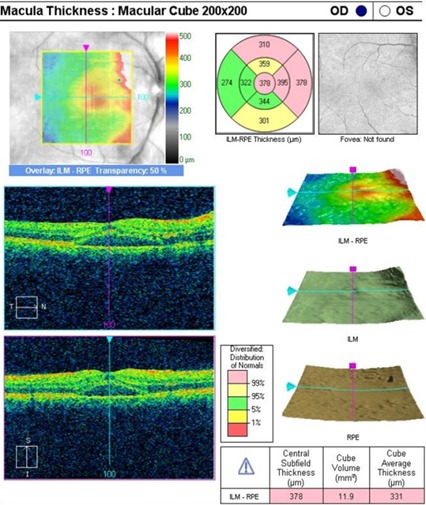
OCT – Macula of the right eye

**Fig. 4:**
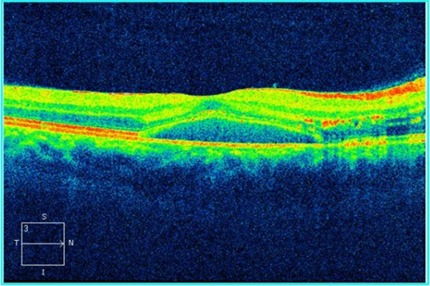
High Definition OCT of the macula of the right eye

**Fig. 5:**
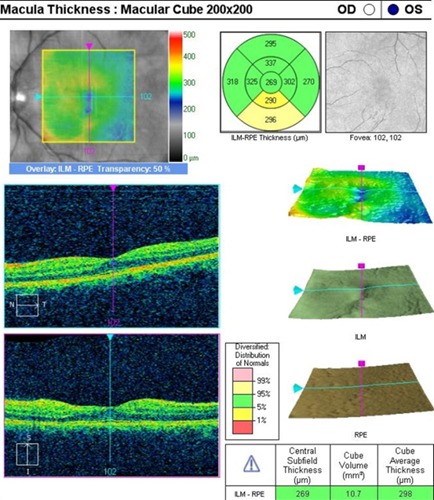
OCT – Macula of the left eye

**Fig. 6:**
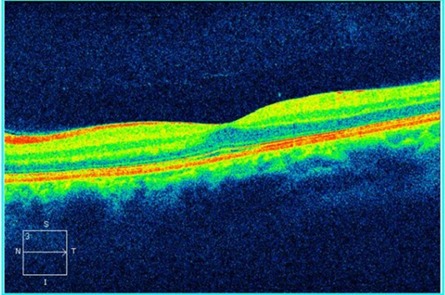
High Definition OCT of the macula in the left eye

An OCT was performed to assess the degree of severity of retinal damage, which, at macular examination, showed (**Fig. 3**,**4**) deformation of the foveal contour in the right eye and increased retinal thickness with neurosenzorial retinal detachment due to the underlying intraretinal fluid accumulation. Although the retinal thickness values parameters were good in the left eye (**Fig. 5**,**6**), a slight increase of the outer plexiform layer thickness was observed due to the accumulation of intraretinal fluid.

**Fig. 7:**
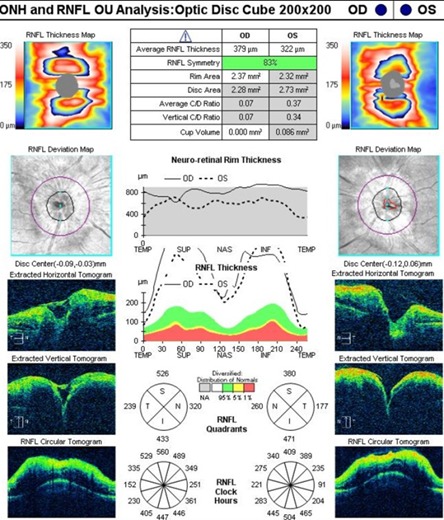
OCT – Optic disc analysis in both eyes

An artefactual increase of the thickness of the retinal nerve fiber layer (RNFL), a blurry margin of the papilla and the significant decrease of the topographic parameters of the excavation were found in the tomographic examination of the optic nerve (**Fig. 7**), all of which induced by the papillary oedema.

Based on the anamnesis, the general clinical examination and the ophthalmological examination, the first stage diagnosis was hypertensive neuroretinopathy in both eyes. Therefore, interdisciplinary complementary consultations were necessary. After the cardiological examination, the diagnoses were: malignant high blood pressure undergoing etiological evaluation, left ventricular hypertrophy and anxious syndrome. The endocrinological, nephrological and diabetes and nutrition disorders examinations were within normal limits.

Paraclinical and laboratory investigations were within normal limits, except for mild hypertriglyceridemia. Echocardiography and electrocardiography confirmed left ventricular hypertrophy, the mark of long-term hypertension.

Imaging investigations were also performed. Abdominal-pelvic ultrasonography showed a nodule in the right adrenal gland, confirmed by computer tomography scan. As there were not enough information about the nodule, a magnetic resonance imaging (MRI) was recommended, but the patient refused it, complaining of anxiety reasons.

Corroborating the data we have obtained up to that moment, the positive diagnosis of hypertensive neuroretinopathy was established in both eyes, the previously mentioned cardiological diagnoses and the nodular formation undergoing etiological evaluation.

The differential diagnosis of the ophthalmological aspect can be done with:

1. Retinal vascular disease, such as:

• Diabetic retinopathy – excluded by normal laboratory tests. 

• Retinal vein occlusion – due to the presence of retinal haemorrhages in the context of an acute condition. It cannot be excluded, being frequently associated. It should also be noted that in retinal vein occlusion, the outer barrier is affected, leading to neurosenzorial retinal detachment. Due to that, if the neurosenzorial retinal application occurs after medical treatment, the diagnosis of hypertensive retinopathy is supported at the expense of retinal vein occlusion [**4**].

2. Other causes of papillary oedema:

• Intracranial hypertension – excluded by the absence of the symptoms and not accompanied by other retinal signs

• Arteritic/ Non-Arteritic Anterior Ischemic Optic Neuropathy – excluded by the good visual acuity and the appearance of the optic nerve disc, aspect that does not suggest inflammatory aetiology

• Infectious/ parainfectious cause – excluded by normal laboratory tests

• Anaemia – excluded by normal laboratory tests

• Polycythaemia – excluded by normal laboratory tests

3. Neuroretinitis:

• Syphilis – excluded by the negative VDRL

The differential diagnosis of the aetiology is between essential and secondary high blood pressure. Secondary high blood pressure can be caused by numerous diseases, such as renal artery stenosis, parenchymal kidney disease, coarctation of the aorta, pheochromocytoma, hyperaldoseronism, etc. [**9**]. Based on the confirmation of adrenal gland nodule, the patient was suspected of pheochromocytoma. Further blood tests and imagistic investigation were needed in order to assess the certain aetiological diagnosis. The patient was followed-up further by the endocrinologist and cardiologist.

The election treatment was of the underlying disease, meaning the high blood pressure. The cardiologist initiated antihypertensive treatment, recommending ramipril, bisoprolol and indapamide, and we considered using nicergoline 30 mg, which is a vasodilator that increases cerebral blood flow, acetylsalicylic acid 75 mg, which prevents thrombus occlusion and arteriolar spasm, relaxing the walls, acetazolamide 250 mg, with a favourable effect on neurosenzorial retinal detachment and retinal oedema, and K+ 39 mg + Mg2+ 12 mg, to compensate the depletion effect of acetazolamide.

The patient returned to the ophthalmological department after three months, when we noted an improvement of the visual acuity in both eyes (20/ 16 S.C.), the disappearance of the metamorphopsia and a normal value of the blood pressure under treatment (110/ 70 mmHg).

**Fig. 8:**
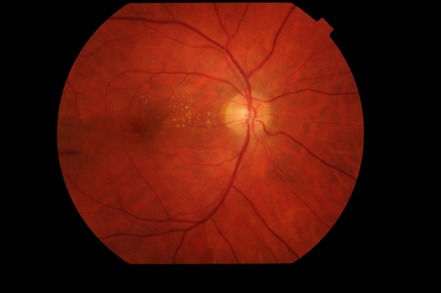
Ophthalmoscopic examination of the right eye after 3 months

Ophthalmoscopic aspect of the right eye was improved (**Fig. 8**). We noticed the remission of the pathological elements: a pink papilla with net edges and visible excavation, the partial remission of the macular exudates and the disappearance of the cotton wools spots.

**Fig. 9:**
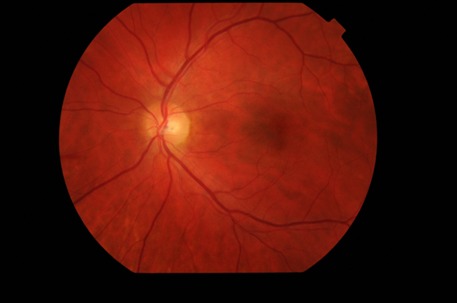
Ophthalmoscopic examination of the left eye after 3 months

The same favourable aspect was found in the left eye (**Fig. 9**).

**Fig. 10:**
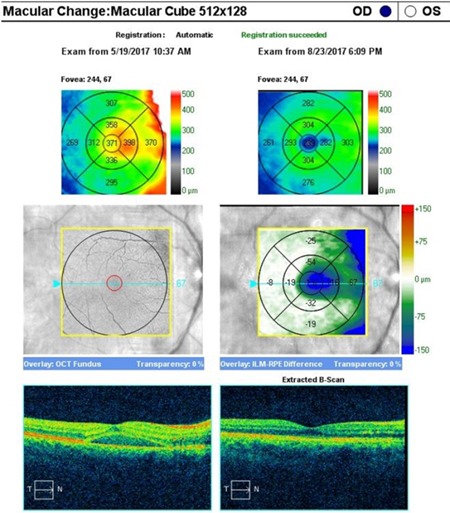
OCT – Macular Change Analysis after 3 months in the right eye

**Fig. 11:**
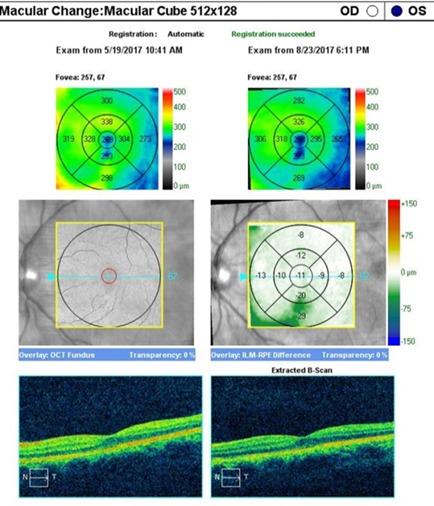
OCT – Macular Change Analysis after 3 months in the left eye

The OCT (**Fig. 10**) revealed a normalisation of the retinal thickness in the right eye, intraretinian fluid resorption and neurosenzorial retinal application. The retinal thickness slightly decreased in the left eye (**Fig. 11**), confirming the subtile intraretinal fluid presence at presentation.

**Fig. 12:**
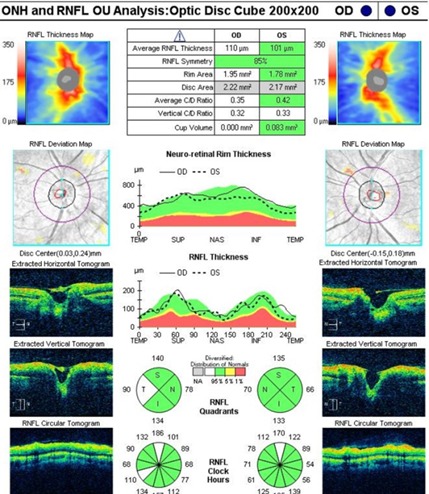
OCT – Optic disc analysis in both eyes after 3 months

**Fig. 13:**
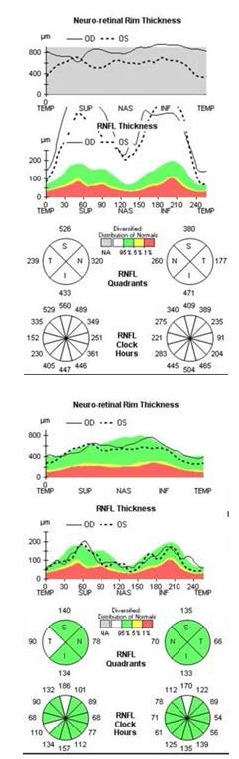
OCT - RNFL evolution (left – at presentation and right – at 3 months)

A normal aspect of it was bilaterally observed on the optic nerve head OCT examination (**Fig. 12**), with the disappearance of the oedema and the normal return to the topographic parameters of the optic disc. A bilateral normal RNFL thickness (**Fig. 13**) was also noticed.

In order to confirm suspicion of pheochromocytoma, the patient had the indication to perform an upper abdominal MRI and to dose catecholamines and their metabolites under basal conditions. If pheochromocytoma was demonstrated, an aimed aetiological treatment was possible, with an optimal blood pressure control and a good long-term vital and ophthalmological prognosis. Invalidation of a secondary cause would lead to reconsidering the high blood pressure as being essential, requiring indefinite pathogenic treatment with reserved long-term vital and ophthalmological prognosis.

## Discussions

Regarding the frequency, it was noted in literature that the low incidence of pheochromocytomas in the hypertensive population was estimated at 0.1%-1%; only 10% of them appearing in young people, the rest being diagnosed starting with the 5th decade [**10**]. The incidence of high blood pressure in young people (18-35 years) is between 9.6% and 11.2%. Of these, only 17.4% have essential high blood pressure, the remaining 82.6% have secondary high blood pressure [**11**].

We noticed many case particularities such as the following:

• Young age of high blood pressure debut

• The ocular complaints have led to medical presentation, although high blood pressure is a systemic disease 

• The correct and complete diagnosis required a multidisciplinary management 

• Patient nonadherence to the antihypertensive treatment prescribed before ophthalmological initial presentation led to the appearance of ocular complications of high blood pressure 

• Poor patient compliance, as he refused MRI investigation due to anxious syndrome, had an unfavorable impact on etiological diagnosing 

• Patient had a favorable evolution under antihypertensive treatment with quasi-integral functional and structural ophthalmological recovery

• Further medical evaluation is needed to assess if there are any other complications of high blood pressure
